# Phlebotomy and quality in the African laboratory

**DOI:** 10.4102/ajlm.v3i1.132

**Published:** 2014-08-18

**Authors:** Henry A. Mbah

**Affiliations:** 1 Senior Technical Advisor Lab, FHI-360, Nigeria

## Abstract

Phlebotomy, the act of drawing blood through venepuncture, is one of the most common medical procedures in healthcare, as well as being a basis for diagnosis and treatment. A review of the available research has highlighted the dearth of information on the phlebotomy practice in Africa. Several studies elsewhere have shown that the pre-analytical phase (patient preparation, specimen collection and identification, transportation, preparation for analysis and storage) is the most error-prone process in laboratory medicine. The validity of any laboratory test result hinges on specimen quality; thus, as the push for laboratory quality improvement in Africa gathers momentum, the practice of phlebotomy should be subjected to critical appraisal. This article offers several suggestions for the improvement of phlebotomy in Africa.

## Introduction

Medical laboratory services, despite playing a pivotal role in Africa’s healthcare system, have suffered gross neglect for decades.^1,2^ Government investment in laboratories is often inadequate, resulting in substandard laboratory infrastructure; poorly-trained and unmotivated staff; and a limited scope of testing services. Furthermore, it is widely understood that insufficient investment in the laboratory can have a negative effect on the quality of testing services, impacting the overall quality of healthcare.

Over the past five years, efforts have been made to improve laboratory services in Africa. In January 2008, the Consensus Meeting on Clinical Laboratory Testing Harmonization and Standardization convened governments, agencies and development partners in Maputo, Mozambique.^3^ The meeting established the Maputo Declaration on Strengthening of Laboratory Systems, aimed at improving clinical laboratory services in developing countries.^3^ Subsequent meetings held in 2008 on African laboratory medicine in Lyon, France, Yaoundé, Cameroon and Dakar, Senegal formulated strategies for laboratory strengthening and arrived at several landmark achievements.^3^ In 2009, the World Health Organization, Regional Office for Africa (WHO-AFRO) and partners launched the Stepwise Laboratory Quality Improvement Process Towards Accreditation (SLIPTA) to help laboratories in resource-limited settings strive toward international accreditation.^3,4^ The African Society for Laboratory Medicine (ASLM) was launched in 2011 and ASLM’s first international conference for laboratory medicine was held in 2012.

### Phlebotomy and the error-prone pre-analytical stage

Phlebotomy, also called venesection or venotomy, is the incision into a vein for the purpose of drawing blood^5^ that is used for laboratory analysis, diagnosis, transfusions and research. The person who performs phlebotomy is called a phlebotomist. Phlebotomists in Africa are also commonly responsible for collecting and properly packaging specimens (blood, sputum, urine, other body fluids, tissues, etc.), accepting incoming specimens and routing specimens to the proper section for analysis. The significant role phlebotomists play in the delivery of essential healthcare services has been described numerous times in existing literature.^6,7,8,9^ Phlebotomists are often the only laboratory professional a patient will meet during a hospital stay.

Accurate and precise laboratory test results depend on properly-performed phlebotomy in order to obtain high-quality specimens. The most well-trained testing staff, using the most sophisticated instruments, cannot produce accurate results from a poorly-collected specimen.

Traditionally, the laboratory testing process is divided into three phases: pre-analytical, analytical, and post-analytical. There have also been suggestions that the pre-analytical phase be divided into a ‘pre-pre-analytical phase’ and a ‘true pre-analytical phase’.^10^ Phlebotomy falls within the realm of the pre-pre-analytical phase, which includes steps (test requesting, patient and sample identification, sample collection and sample transportation) that may neither be performed in the laboratory nor undertaken by laboratory personnel. The post/pre-analytical phase, which is carried out in the laboratory after specimen reception, involves the steps required to prepare samples for analysis (centrifugation, aliquoting and sorting).

Several studies suggest that in laboratory diagnosis, most errors occur within the pre-analytical phase (46% – 70%), followed by the post-analytical phases (18% – 47%), with the fewest errors occurring in the analytical phase (7% – 13%).^11,12,13^ The most frequent pre-analytical errors in laboratory medicine include: missing sample and/or test request; incorrect or missing identification; *in vitro* haemolysis; undue clotting; use of the wrong container; contamination from infusion route; insufficient sample; inappropriate blood-to-anticoagulant ratio; insufficient mixing of the sample; inappropriate transport; and incorrect storage conditions.^12^

### Phlebotomy has been undervalued worldwide

Historically, the critical role of phlebotomy has been overlooked,^14^ having been suggested as the most underestimated procedure in healthcare.^6,14,15^ For example, although most employers in the United States (US) require valid certification or a licence issued by an accredited phlebotomy training programme or a professional body such as the American Society of Phlebotomy Technicians (ASPT), only five US states mandate phlebotomy certification for practise.^16^

According to a recent survey on phlebotomy practice in 28 European countries, 21% of the countries do not require specific training for phlebotomy; national phlebotomy guidelines are available in only 25% of the countries; and only 36% have specific training available as a continuous educational resource.^17^ In many countries (and most countries in Europe), there are no professional phlebotomists.^13^ Phlebotomy is performed by doctors, nurses, laboratory staff and other healthcare professionals.

### Current practice in Africa

Anecdotal evidence (i.e., accounts from individual healthcare workers) from countries such as Cameroon, Chad, Côte d’Ivoire, Kenya and Nigeria indicates a high prevalence of suboptimal phlebotomy practices. Unfortunately, because of the paucity of published information on phlebotomy practice in Africa, this paper refers to information from Europe and the US, where causes of phlebotomy service issues have been well researched and may be similar to those faced in resource-limited settings.

#### Staff

In many facilities, laboratory phlebotomy staff draw blood from outpatients whereas doctors and nurses usually draw blood from inpatients. Less oversight from the laboratory could contribute to service quality issues if medical staff have multiple tasks to perform simultaneously. Use of trained phlebotomy-specific personnel may greatly reduce pre-analytical error rates.

In many facilities across Africa, resource constraints require that staff be cross-trained for multiple tasks, possibly eroding specific skills and resulting in excessive workloads. Furthermore, the practice of rotating staff through different facilities likely decreases institutional expertise, especially if adequate planning and training are not performed well in advance.

A study in Europe found that the rates of pre-analytical errors are higher for inpatients than outpatients, for whom procedures are performed by personnel under direct laboratory control.^18^ A publication from the US concluded that phlebotomists are preferred over nurses and physicians for blood draws.^19^ Increasingly, phlebotomy skills are being diluted in African healthcare settings through the implementation of task shifting and multi-skilled staffing strategies. Thus, a decrease in phlebotomy expertise is exposing an increasing number of facilities to serious underlying safety problems and the likelihood of liabilities.

#### Space

In most cases, dedicated space is not available for specimen collection; blood is drawn in patient waiting areas, laboratory result-collecting areas, in the heart of the laboratory, or in corridors and passageways, without demarcation. Thus, confidentiality may be compromised.

#### Quality of specimen

The correct order of blood draw is not well understood and recommended volumes are not always considered.^20^ Blood may be drawn from intravenous infusion devices and needles may be withdrawn with the tourniquet still in place. There may also be inadequate quality checks by supervisors.

#### Logistics

Materials and supplies are often inadequate; blood is frequently drawn into an ordinary syringe and moved, exposed, from the wards to the laboratory. Using ordinary syringes, the volume and proportion of mixture with anticoagulant and other additives relative to the intended test may be ignored. In the wards in particular, piercing the skin with ordinary needles and failure to put pressure on the puncture site, have most likely caused risky situations in which blood leaks down the patient’s arm onto the bed and clothing.

#### Identification

Poor labelling is a major source of concern.^20^ Labelling is done locally, by matching the patient name with a number written with wax marker, coloured pencil, or marker onto the tubes and, in some cases, on the rubber tube caps only. This may lead to a mix-up of specimens during processing when, in some cases, the tops are removed before centrifugation. Some technicians may claim they still know which tube belongs to which patient, which is highly unlikely. Transfusion-related deaths^21^ and undue surgeries^22^ have been traced back to patient- or specimen-identification errors. One laboratory even reported pregnancy in a male patient.

#### Safety

Some staff members may not wear or change gloves^23^ between patients, either because of limited supply or lack of training and supervision. In some cases, staff members have expressed concern that they would not be able to feel a vein with gloves on. Touching the incision site to find the vein after alcohol swabbing is common^23^ and can expose the patient to risk of infection.

Sharps containers and colour-coded waste bins are often absent; it is not uncommon to find only a single trash bin without lining for general use. Such practices have exposed healthcare workers to needle-stick injuries and sharps injuries.^24,25^

#### Documentation

Standard operating procedures (SOPs) are not readily available. Where they exist, SOPs are too often locked up and not easily accessible to all staff members who perform phlebotomy. In the wards, clinical SOPs are severely lacking. Policies are rarely available and job aids are uncommon, or there is no appropriate place to affix them.

#### Training

Special training, continuous and refresher training and certification or competency assessment in phlebotomy are rarely mandatory. It is worth noting that in South Africa, continuous phlebotomy training and certification is required.

In donor-funded antiretroviral therapy (ART) programmes, the drive to put as many persons as possible on ART may overshadow plans for phlebotomy training. Ironically, the quality of an ART programme hinges on the entry point of sample collection and testing.

### Existing complexity in the setup

Although the importance and vulnerability of the pre- and post-analytical phases have been acknowledged for many years, current quality management programmes still tend to focus on the performance and efficiency of analytical processes and activities within the direct control of the laboratory.

There are concerns on the thoroughness of coverage of the pre-analytical phase in the major accreditation schemes. The current tiered quality improvement scheme, SLIPTA, focuses on resource management covering nine of the 14 requirements considered by ISO 15189/2007 on pre-examination procedures.^26^ Despite the coverage by ISO 15189/2007, pre-analytical errors remain, even in an accredited laboratory.^27^ To date, the pre-analytical variables that lie outside the direct control or supervision of laboratory personnel are difficult to monitor, as tools and policies are not fully standardised or harmonised worldwide.

### Recommendations

Some suggestions are proposed to stimulate thoughts and actions that will help to improve phlebotomy practice and reduce pre-analytical errors in laboratory medicine. Firstly, clear written procedures from existing guidelines should be developed.^28,29^ Phlebotomy techniques should be standardised and SOPs, operative guidelines and preventive and reporting policies widely disseminated. Secondly, a dedicated phlebotomist should be appointed, if possible, and/or specific healthcare professional training, continuous education and routine competency assessment should be enhanced. Quality indicators focusing on the pre-analytical phase should be adapted, implemented and monitored in quality improvement projects.^30^ External quality assurance programmes should be modified to check the entire examination process, including pre-analytical and post-analytical procedures. Phlebotomy services in health facilities should be centralised and communication amongst healthcare professionals and interdepartmental cooperation should be improved. Finally, field studies should be undertaken in order to address the dearth of research in the area of phlebotomy practice and pre-analytical errors in Africa.

## Conclusion

In some African countries, the quality of phlebotomy, the entry point to laboratory testing, is inadequate. This important period in the struggle for laboratory quality improvement in Africa provides a window of opportunity to enhance strategies to improve phlebotomy practices.
